# Fertility preservation with successful pregnancy outcome in a patient with transplanted heart and non-Hodgkin’s lymphoma – a case report

**DOI:** 10.1186/s12884-019-2587-x

**Published:** 2019-11-19

**Authors:** Ana Sofia Pais, Nuno Guerra, Daniela Couto, Ana Paula Sousa, Teresa Almeida-Santos

**Affiliations:** 1 Reproductive Medicine Unit, Centro Hospitalar e Universitário de Coimbra (CHUC), Praceta Prof. Mota Pinto, 3000-075 Coimbra, Portugal; 2 Obstetrics A Unit, Centro Hospitalar e Universitário de Coimbra, Coimbra, Portugal; 3 Faculty of Medicine, University of Coimbra, Coimbra, Portugal; 4 Center for Neuroscience and Cell Biology, University of Coimbra, Coimbra, Portugal

**Keywords:** Pregnancy, Oocyte cryopreservation, Pelvic irradiation, Heart transplant

## Abstract

**Abstract:**

Background: Fertility preservation must be discussed with reproductive age women before cancer treatment. Heart transplantation raises complex issues in pregnancy. Pregnancy in a heart transplant woman after pelvic irradiation involves close multidisciplinary follow-up to avoid complications in the mother and the foetus. We report the first live birth in a heart transplant woman after pelvic irradiation, chemotherapy and fertility preservation.

Case presentation: A 36-year-old heart transplant woman with pelvic non-Hodgkin lymphoma spared her fertility, with cryopreservation of oocytes and embryos, before chemotherapy and pelvic irradiation. After multidisciplinary discussion and pre-conception evaluation, pregnancy was achieved. A close follow-up by a multidisciplinary team allowed a normal pregnancy without maternal or foetal complications and the delivery of a healthy infant.

**Conclusions:**

Achieving pregnancy in heart transplant women with iatrogenic ovarian failure after oncologic treatment including pelvic irradiation is possible and can be successful. Careful and close surveillance by a multidisciplinary team is mandatory due to increased risk of maternal and foetal complications.

## Background

A growing number of women have an oncological diagnosis before ending or even starting their reproductive project. Nevertheless, many cancers are curable, so the quality of life after cancer needs to be addressed, as the risk of impairing gonadal function is high [1]. Fertility preservation treatments give hope for a successful pregnancy once the disease is overcome, but individualized reproductive counselling is mandatory both before and after cancer treatment [1, 2].

As well as premature ovarian failure, previous pelvic irradiation is associated with smaller uterine volume, which can be related to direct damage and/or hormonal depletion [2]. However, available evidence comes from radiation exposure during childhood or adolescence, and it is not known if it can be extrapolated to adult women that undergo pelvic irradiation [2].

On the other hand, fertility and pregnancy in heart transplant patients raise complex issues, considering the high risk for potential maternal and foetal complications [3]. Since the first successful pregnancy after heart transplantation in 1988, more than 12,000 heart transplants have been performed in women, with a 5-year patient survival of 69%, raising the issue of developing appropriate pregnancy management strategies [4]. For non-Hodgkin lymphoma, the 5-year survival rate is 71%. However, the 5-year survival rate vary widely for different types and stages of lymphoma, being 51,1% for a stage IV large B-cell lymphoma [5].

In this case report, we describe a successful pregnancy and delivery after fertility preservation in a heart transplant woman after pelvic lymphoma radiation. This is a unique case as it combines the challenge of pregnancy in a heart transplant patient under immunosuppression, with fertility preservation and the consequences of oncological treatments, namely pelvic radiotherapy. Informed consent was obtained from the patient for this report and approved by the Hospital Ethics Committee.

## Case presentation

In 2006, a 25-year-old woman underwent heart transplanted due to dilated cardiomyopathy of unknown aetiology. She was under regular follow-up and treatment in the Cardiothoracic Surgery Unit, without rejection. The patient was previously healthy and had no family history, with 18,96 Kg/m^2^ of body mass index.

Seven years later, a pelvic tumor of 14 × 10 cm was seen in a computerized tomography scan, involving the uterus and adnexal regions, with another mass of 6 × 5 cm involving the right colon. Laparoscopic biopsies were performed and revealed a stage IV non-Hodgkin’s lymphoma, more precisely diffuse large B-cell lymphoma.

As the woman wished to spare her fertility potential, ovarian stimulation was started before oncological treatment. After collection of 12 mature oocytes, 6 were vitrified and another 6 were fertilized and cryopreserved at the 2PN stage (pre-zygotes).

Immediately after oocyte collection, chemotherapy was initiated with 8 cycles of R-CHOP (rituximab, cyclophosphamide, doxorubicin, vincristine, prednisone), with pegylated liposomal doxorubicin (total dose 800 mg) in order to avoid cardiotoxicity. Due to residual mass in a positron emission tomography scan, pelvic radiotherapy was initiated (36 Gy/18 fraction, in abdominal lymph nodes). At the end of therapy (May 2014), a complete remission was achieved without cardiac toxicity.

After oncological treatment, the woman became amenorrhoeic, with genital atrophy. Atrophic ovaries, uterus and endometrium were seen in the ultrasound scan. The hormonal analysis confirmed the diagnosis of premature ovarian failure with an elevated follicle stimulating hormone level, on two occasions more than 1 month apart (122 and 137 mUI/mL), with low oestradiol (< 12 pg/mL) and anti-Müllerian hormone levels (< 0.0004 pg/L).

As the couple wished for a pregnancy, a multidisciplinary discussion, including cardiothoracic surgery and haemato-oncology, was conducted, as well as a pre-conception evaluation. Two years after the end of oncological therapy, endometrial preparation was initiated to allow embryo transfer. Endometrial preparation was started with oral oestradiol 6 mg per day, however endometrial ultrasound evaluation was unsatisfactory. In subsequent endometrium preparation attempts, sildenafil and vitamin E were unsuccessfully associated. After five months of failed attempts, the combination of 6 mg oral oestradiol daily plus 100 mg transdermal weekly achieved an adequate endometrium (7 mm tri-layered endometrium on ultrasound) and then vaginal progesterone (400 mg 3 times daily) was started. Cryopreserved 2PN oocytes were thawed and one resulting embryo was transferred after five days after progesterone onset.

After achieving pregnancy, the patient was followed regularly by a multidisciplinary team including a cardiologist and an obstetrician. She had also regular follow-up by cardiothoracic surgery and haemato-oncology.

At the first appointment, the therapeutic regimen included immunosuppressive drugs (tacrolimus 3 mg, prednisolone 4 mg), anticoagulant medication (enoxaparin 40 mg), platelet aggregation inhibitor (indobufen 200 mg), calcium antagonist drug (diltiazem 60 mg), diuretic therapy (furosemide 20 mg), proton pump inhibitor (pantoprazole 40 mg=) and statin (pravastatin 20 mg), plus pregnancy supplementation with folic acid 5 mg and the endometrial preparation regimen (oestradiol 8 mg oral and 100 mg transdermal and progesterone 1200 mg vaginal). The patient was informed of the teratogenicity of her medication and the risks of suspending it. The medication was progressively reduced so that in the third trimester we achieved the combination of immunosuppressive drugs (tacrolimus 3 mg, prednisolone 4 mg), anticoagulant medication (enoxaparin 40 mg), platelet aggregation inhibitor (acetylsalicylic acid 100 mg, stopped at 35 weeks), calcium antagonist drug (diltiazem 60 mg), diuretic therapy (furosemide SOS), plus pregnancy supplementation with ferrous sulphate 90 mg.

Screening for infections and gestational diabetes were negative with normal renal and hepatic function. Blood pressure was normal and no proteinuria was detected during pregnancy.

Foetal ultrasound examination at 12 weeks was normal, with nuchal translucency below the 95th percentile, and the first trimester combined screening of aneuploidies was negative. Ultrasound at 22 weeks revealed a normal foetal morphology, except left clubfoot, normal heart evaluation, and normal growth (biometry in the 50th percentile). According to Fetal Medicine Foundation charts [6], at 30 weeks foetal biometry was in 94th percentile (1865 g), confirmed at 34 weeks (2827 g), with bilobed fundal placenta, with no signs suggestive of placenta accreta.

The maternal echocardiography performed in third trimester of pregnancy revealed normal left ventricular function (54%).

**Figure 1:**
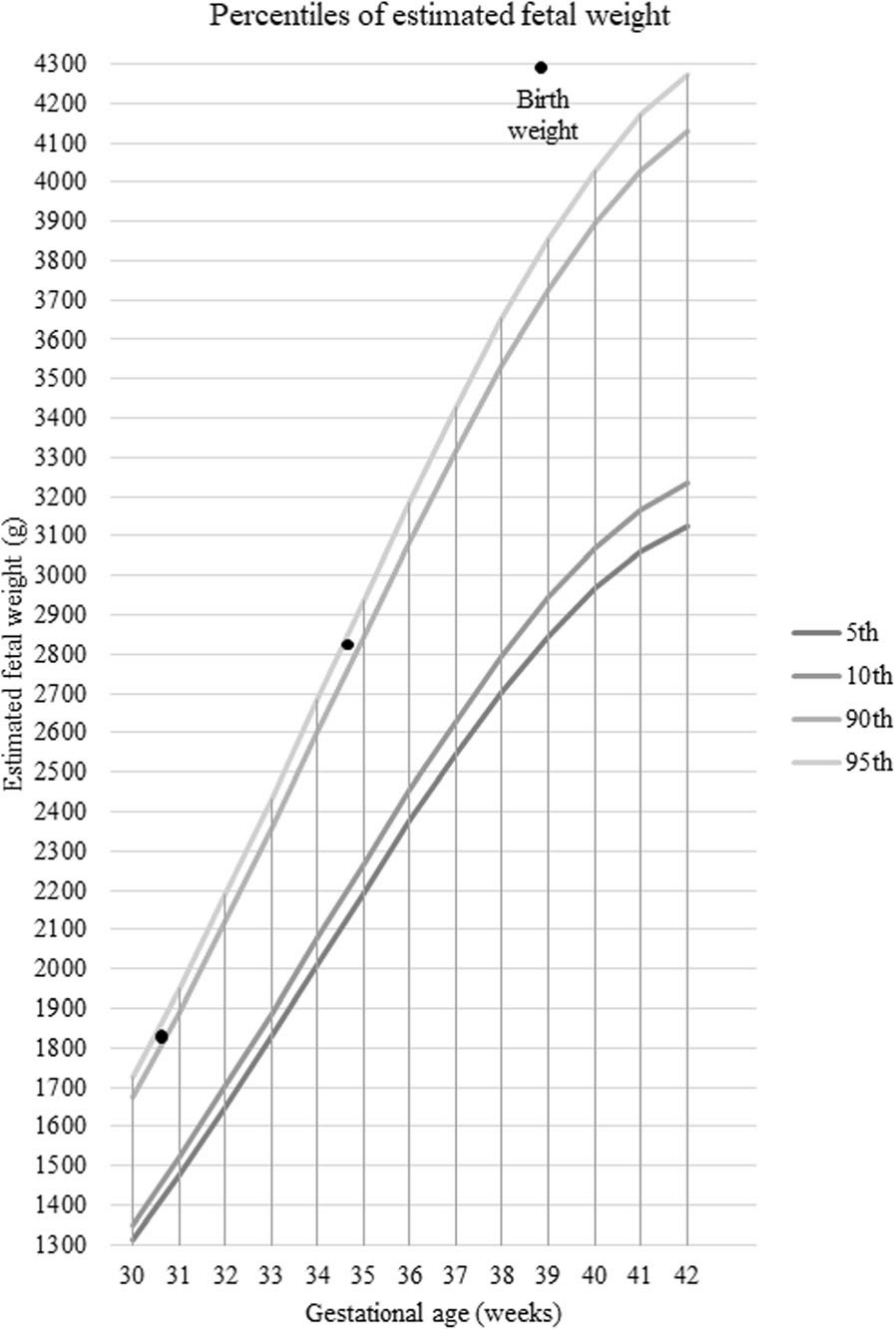
Estimated foetal weight chart**.** Ultrasonography performed at 30 weeks and 5 days revealed an estimated foetal weight at the 94rd percentile, which was maintained in the growth assessment performed 4 weeks later. Percentile chart adapted from Fetal Medicine Foundation [6]

At 39 weeks of gestation, a caesarean section was performed, due to suspected foetal macrosomia (Fig. 1). She delivered a female infant of 4305 g, APGAR score 9/10/10. Prophylactic antibiotic therapy for caesarean section was administered. Surgery was done under epidural anaesthesia without incidents, except for moderate bleeding, due to difficulty in manual removal of placenta, although no signs of placenta accreta were seen in the histological analysis.

She breastfed the newborn and no neonatal complication were reported. No severe graft dysfunction or infection were observed during the puerperal period and she was discharged 3 days later.

## Discussion and conclusions

With improving oncological treatments, quality of life in cancer survivors is of growing significance. Loss of fertility is a key issue for young female cancer survivors, depending on the follicular reserve, the age of the patient and the type and dose of drugs used. As observed in the case reported, a high risk for premature ovarian failure is found after gonadotoxic treatment and pelvic radiation [1].

Ovarian stimulation and oocyte or embryo cryopreservation prior to chemoradiation must be discussed with every young female cancer patient. However, there is scarce experience in the management of women who wish to get pregnant following pelvic radiation [1, 2]. Sex steroid replacement, in a progressively escalating dose regimen, may have a role in the restoration of satisfactory uterine volume, endometrial thickness, and uterine vascularization in women who have been exposed to lower doses of uterine radiation (< 25 Gy) at a post-pubertal age [2].

Preconception evaluation is crucial in heart transplant recipients who wish to become pregnant, including counselling, appropriate evaluation of graft function, and monitoring of immunosuppressive agents [4]. Regarding previous pelvic irradiation, there is minimal data in the literature about fertility and pregnancy outcome of women exposed in adulthood [2]. The threshold radiation dose for uterine damage to occur so that pregnancy is not sustainable is unknown, but Teh et al. (2014) suggest that patients receiving > 45 Gy during adulthood should be counselled to avoid attempting pregnancy [2]. Appropriate counselling with regard to the ability of the irradiated uterus for carrying a pregnancy should be provided to these women. A successful pregnancy will require not only a viable embryo but also a uterine cavity that is receptive to embryo implantation and a uterus that has the ability to accommodate normal growth of the foetus to term [2].

Achieving pregnancy after pelvic irradiation in a heart transplant patient needs close monitoring by a multidisciplinary team [4, 7].

Pregnancy and postpartum are periods of increased risk of complications in post-cardiac transplant patients [4]. Risks include pregnancy induced hypertension (hypertension, pre-eclampsia, eclampsia), infection, gestational diabetes and thromboembolic disease (venous thromboembolism, pulmonary embolism), all of which can lead to adverse maternal, foetal, and neonatal outcomes [3, 4]. The most common maternal complication in the pregnant heart transplant patient is hypertension [4, 8]. The incidence of preeclampsia in cardiac recipients is 18%, higher than the 2 to 7% in healthy nulliparous women [8]. Pre-eclampsia increase the risks of foetal growth restriction, low birth weight, and preterm delivery [4]. For pregnant heart transplant patients, the risk of spontaneous abortion is 15 to 20% [4]. Graft rejection is another possible complication, reported in 11% during pregnancy and 6% during the first 3 months after delivery [8].

Pelvic irradiation also increases the risk for pregnancy-related complications, including spontaneous miscarriages, preterm labor and delivery, low birth weight, placental abnormalities and uterine rupture [7]. These findings have been attributed to reduced uterine volume, impaired uterine distensibility due to myometrial fibrosis, uterine vasculature damage, and endometrial injury [2, 7]. Placental attachment disorders, including placenta accreta or percreta, are related to endometrium injury that prevents normal decidualization. It has also been hypothesized that radiation therapy may lead to diffuse thinning of the myometrium, increasing the risk of uterine rupture [7].

First pregnancy after chemo-immuno-radiation therapy for a pelvic lymphoma was described in 2008, Ferreri et al. described a spontaneous pregnancy with a vaginal delivery of a healthy baby 36 months after pelvic radiotherapy [9]. The therapeutic strategy was similar, but in a lower dose (6 versus 8 cycles of R-CHOP and 30-6Gy versus 36Gy of radiotherapy). For fertility preservation, they used ovary transposition. This technique is relatively simple and particularly interesting before pelvic radiotherapy, however it is underused as it does not protect from the gonadotoxic effect of systemic treatment as chemotherapy.

Birth defects may originate through multiple mechanisms and may be caused by a variety of possible exposures, including medications in early pregnancy. The history of chemotherapy and pharmacological treatment at the time of the first consultation could be a concern in this case. However, a recent meta-analysis and systematic review report that the most common and clinically relevant risk factors for club foot are family history, selective serotonin reuptake inhibitors, amniocentesis, maternal/paternal smoking, maternal obesity, gestational diabetes. None of these risk factors were present in the clinical case presented [10]. Vaginal delivery is the recommended method of delivery in heart transplant recipients [4]. A caesarean section should be performed for obstetric indications [3] and is reported in 40% of deliveries in this population [8]. In the reported case, a caesarean section was performed due to suspected foetal macrosomia, under epidural anaesthesia, in order to reduce sympathetic responses induced by pain and acute fluctuations of blood pressure during labour [3].

Prophylactic antibiotic therapy is not routinely recommended, except for high risk condition [3, 4, 11], and in this case it was administered to prevent subacute bacterial endocarditis and post-operative infection. During labour, arrhythmias were closely monitored via continuous electrocardiogram, as recommended [4]. Because of the haemodynamic changes and volume shifts that occur immediately post-delivery, the cardiac transplant patient is at highest risk during the immediate postpartum period [4].

Breastfeeding has typically been discouraged in heart transplant.

women, as all immunosuppressive medications are secreted through breast milk and long-term effects of immunosuppressive drug exposure on infants are unknown [3]. However, recent studies have shown that transplant recipients taking prednisone and tacrolimus should not be discouraged from breastfeeding [12].

All women, including heart transplant patients, should be counselled for fertility preservation in case they developed oncological disease. The strategies of fertility preservation are not different for heart transplant patients. When choosing the best method for fertility preservation a multidisciplinary team should consider the patients’ age, pubertal status and maturity level, cancer type, prognosis, staging and existence/risk of metastasis, time available for fertility preservation intervention before initiating treatment and the indication and limitations for each fertility preservation method applied to each specific patient. Options for fertility preservation in cancer patients include oocyte and embryo cryopreservation as established methods and ovarian tissue cryopreservation, in vitro maturation of oocytes and artificial ovary as experimental methods [13].

For cases such as the reported, pre-pregnancy risk assessment and counselling should be offered. Subsequently, prenatal surveillance should be performed in specialized centres by a multidisciplinary pregnancy heart team, with at least one monthly consultation [14].

In conclusion, achieving a successful pregnancy in a woman with iatrogenic premature ovarian failure due to oncological treatments, including pelvic radiation and a previous heart transplant is a conquest of modern medical management and team work. Additionally, a history of cardiac transplantation is not a contraindication for fertility preservation if those patients developed a curable oncological disease. Proper counselling, careful and close surveillance by a multidisciplinary team is mandatory for successful outcome, due to increased risk of maternal and foetal complications.

## Data Availability

The datasets generated and/or analysed during the current study are not publicly available due to individual privacy but are available from the corresponding author on reasonable request.

## Abbreviations

CHUC: Centro Hospitalar e Universitário de Coimbra
PN: Pre-zygotes
R-CHOP: Rituximab, cyclophosphamide, doxorubicin, vincristine, prednisone
